# Chromosome-level genome assembly of the aphid *Megoura crassicauda*

**DOI:** 10.1038/s41597-025-05328-7

**Published:** 2025-06-13

**Authors:** Tianyu Huang, Xu Cheng, Bing Wang, Guirong Wang

**Affiliations:** 1 State Key Laboratory for Biology of Plant Diseases and Insect Pests, Institute of Plant Protection, Chinese Academy of Agricultural Sciences, Beijing, 100193 China; 2 Kunpeng Institute of Modern Agriculture at Foshan, Shenzhen Branch, Guangdong Laboratory of Lingnan Modern Agriculture, Agricultural Genomics Institute at Shenzhen, Chinese Academy of Agricultural Sciences, Shenzhen, 518124 China; 3 Shenzhen Branch, Guangdong Laboratory for Lingnan Modern Agriculture, Synthetic Biology Laboratory of the Ministry of Agriculture and Rural Affairs, Agricultural Genomics Institute at Shenzhen, Chinese Academy of Agricultural Sciences, Shenzhen, China

**Keywords:** Agriculture, DNA sequencing, Bioinformatics

## Abstract

*Megoura crassicauda* (Hemiptera: Aphididae) is a major pest species that inflicts significant damage on various legume crops worldwide, causing substantial global economic losses. In this study, we present a chromosome-scale genome assembly of *M. crassicauda*. By integrating PacBio long-read sequencing, Illumina short-read sequencing, and Hi-C scaffolding techniques, we constructed a genome assembly spanning 424.45 Mb genome. Approximately 93.74% of the assembly was successfully anchored into five scaffolds, with contig and scaffold N50 reaching 5.55 Mb and 103.55 Mb, respectively. The genome completeness, as evaluated by BUSCO, achieved a completeness score of 97.75%. Additionally, a total of 14,717 protein-coding genes were identified. This high-quality genome assembly of *M. crassicauda* serves as a valuable genomic resource, facilitating further studies into the ecological adaptations of *M. crassicauda* and the development of pest control strategies.

## Background & Summary

Legumes represent one of the most vital crop families in agriculture^1^, serving as the primary source of vegetable protein for both human consumption and livestock feed. However, the productivity of key leguminous crops, such as pea (*Pisum sativum*), broad bean (*Vicia faba*), and faba bean, is significantly threatened by pest infestations^2^. Among these pests, *Megoura crassicauda* (Hemiptera: Aphididae) is a predominant legume pest, and specifically infests leguminous plants, including pea (*P. sativum*), broad bean (*V. faba*), and other *Vicia* spp.^3^, using piercing-sucking mouthparts to extract phloem sap. Its feeding behavior causes substantial agricultural damage, primarily through leaf distortion, stunted growth, and yield reduction, with the most severe impacts occurring during reproductive stages (e.g., flowering and pod development). Native to east Asia (east Siberia, China, Japan and Korea)^4^, *M. crassicauda* has recently been detected in New South Wales, Australia^5^, raising concerns about its potential for global spread. However, despite its significant economic impact and invasive potential, genomic resources for *M. crassicauda* remain limited.

Aphid release droplets from their cornicles when attacked^6^. These droplets contain alarm pheromones that trigger avoidance behaviors in nearby conspecifics, ultimately increasing the survival rate of the aphid population. Although the chemical signal recognition mechanisms of aphid alarm pheromones have been extensively studied, significant knowledge gaps remain regarding the key genes and regulatory mechanisms of their biosynthetic pathways, with interspecies divergence observed in biosynthetic strategies^7^. Notably, aphids of the genus *Megoura* produce alarm pheromones composed of diverse terpenoid compounds^8^, making them an ideal model for investigating the evolution and adaptive divergence of pheromone synthesis pathways. However, the lack of high-quality genomic data for *Megoura* species has hindered cross-species comparative genomic analyses, severely impeding the elucidation of the molecular basis of pheromone diversity.

Since the landmark sequencing of *Acyrthosiphon pisum*^9^, genomic resources for aphids have expanded to 27 aphid species (as recorded in the InsectBase v2.0^10^), spanning destructive pests such as *Myzus persicae*^9^, *Aphis glycines*^11^, *Aphis gossypii*^12^. These datasets provide foundational resources for deciphering the molecular basis of aphid adaptation and stress resistance. However, the current genomic coverage of aphid species remains below 1%, which highlights the critical need to accelerate functional genomics research through omics-driven sequencing initiatives.

Here, we present a high-quality chromosome-scale genome assembly of *M. crassicauda*, achieved through the integration of PacBio sequencing, Illumina sequencing, and chromatin conformation capture (Hi-C) techniques. Gene structures were annotated based on a combination of transcriptomic data, *ab initio* predictions, and homology-based approaches. Species tree was constructed to elucidate the evolutionary relationship of *M. crassicauda* with other Aphididae species. This genome assembly serves as a valuable resource for advancing molecular biology research and developing pest control strategies for this species.

## Methods

### Sample preparation and genomic sequencing

The *M. crassicauda* colony in this study was originally collected from bean fields at the Langfang Experimental Station of the Chinese Academy of Agricultural Sciences. Aphids were maintained on broad bean (*Vicia faba*) plants in a greenhouse under ambient light conditions, with temperature controlled at 20 ± 2 °C and relative humidity maintained at 75%. To establish a colony population of parthenogenetic females, a single female was isolated from the parental colony to establish a new colony. Through five consecutive generations of clonal propagation - with one offspring systematically selected from each generation to initiate the subsequent colony - we established a genetically stable, all-female lineage. This fifth-generation clonal colony, exclusively comprising parthenogenetically reproducing females, served as the experimental material for whole-genome sequencing analyses.

For PacBio sequencing, genomic DNA was extracted from 40 wingless parthenogenetic adult females. Two single-end libraries with 20-kb insert sizes were prepared using the PacBio’s Single-Molecule Real-Time (SMRT) sequencing technology (Pacific Biosciences). Sequencing was performed on the PacBio Sequel II platform, yielding raw reads from a single cell. After quality control, 133.11 Gb of high-quality SMRT sequences were retained, providing ~314 × coverage with an average read length of 12.35 kb (N50 = 17.48 kb). For Illumina short-read sequencing, DNA was extracted from around about 40 wingless parthenogenetic female adults. A 400-bp paired-end library was constructed following standard Illumina protocols and sequenced on the HiSeq X Ten platform, producing 29.35 Gb of paired-end reads with 150 bp length. To enable chromosome-level assembly, a Hi-C library was prepared using established methods^13^. Fresh tissue samples from 40 adult individuals were crosslinked with paraformaldehyde to capture interacting DNA segments. The crosslinked material was digested with DpnII restriction enzyme, and biotinylated nucleotides were used to label the restriction fragment ends. The Hi-C library was quantified and sequenced on the Illumina NovaSeq/MGI-2000 platform, generating ~58.39 Gb of paired-end clean reads with 150 bp length.

### RNA sequencing

Total RNA was extracted from 50 parthenogenetic adult females divided into five biological batches (10 adults per batch) using TRIzol reagent (Invitrogen, Carlsbad, CA, USA)^14^. RNA extracts from all batches were pooled and resuspended in RNase-free water. RNA integrity was assessed by 1% agarose gel electrophoresis, and purity (A260/A280 ratio ≥ 1.8) and concentration were quantified using a NanoDrop ND-2000 spectrophotometer (Thermo Fisher Scientific, Waltham, MA, USA). High-quality RNA samples were selected for cDNA library preparation^15^. Sequencing was performed on the Illumina NovaSeq 6000 platform (Illumina, San Diego, CA, USA) using a 200 bp paired-end strategy. This process yielded a total of 148,198,723 high-quality clean reads, with a Q30 scores exceeding 90%.

### Genome assembly

Quality control of the raw Illumina reads was performed using FASTP v0.20.0^16^. Clean reads were subsequently analyzed with JELLYFISH v2.3.0^17^ to generate a 17-mer frequency distribution map. Genome size estimation was conducted through k-mer spectrum analysis using Genomescope v1.0^18^, revealing a predicted genome size of 428.28 Mb for *M. crassicauda*.

For contig assembly, PacBio reads were initially error-corrected using FALCON v1.8.7 (reads_cutoff: 1k, seed_cutoff: 33k). The corrected reads were then assembled into a draft genome using SMARTDENOVO v1.0 with parameters -J 3000 and -k 19^19^. To improve assembly accuracy, PacBio reads were aligned to the draft genome using BLASR v5.1^20^, followed by one round of genome polishing with ARROW v2.2.2 under default parameters. For further refinement, Illumina reads were mapped to the assembly using BWA v0.7.12^21^, and four iterations of contig polishing were performed with NextPolish v1.0.5 using default parameters^22^. The final contig-level assembly achieved a total length of 424.45 Mb, closely matching the estimated genome size, and achieved a contig N50 of 5.55 Mb (Table 1).

**Table 1:** Major indicators of the *Megoura crassicauda* genome.

Features	Statistics
Estimated genome size (bp)	428,278,444
Assembly size (bp)	424,451,851
Contigs N50 (bp)	5,554,344
Scaffolds number	544
Scaffolds N50 (bp)	103,551,894
BUSCO genes	C: 97.75% [S: 93.81%, D: 3.94%], F: 0.28%
Number of protein-coding genes	14,717

### Hi-C scaffolding

Raw sequencing reads containing low-quality bases (Phred score < 20), short fragments (<30 bp), or adapter contamination were removed using FASTP (v0.20.0) with default parameters. Clean reads were subsequently aligned to the contig assembly using BOWTIE2 v2.3.2 in end-to-end, -very-sensitive -L 30^23^. Valid interaction paired reads were identified using HI-C PRO v2.8.1^24^ with default parameters, while reads with multiple hits and singleton reads were excluded. To cluster, order, and orient the contigs, LACHESIS^25^ was employed with the following parameters: CLUSTER MIN RE SITES=100; CLUSTER MAX LINK DENSITY=2.5; CLUSTER NONINFORMATIVE RATIO=1.4; ORDER MIN N RES IN TRUNK=60; ORDER MIN N RES IN SHREDS=60.

**Fig. 1:**
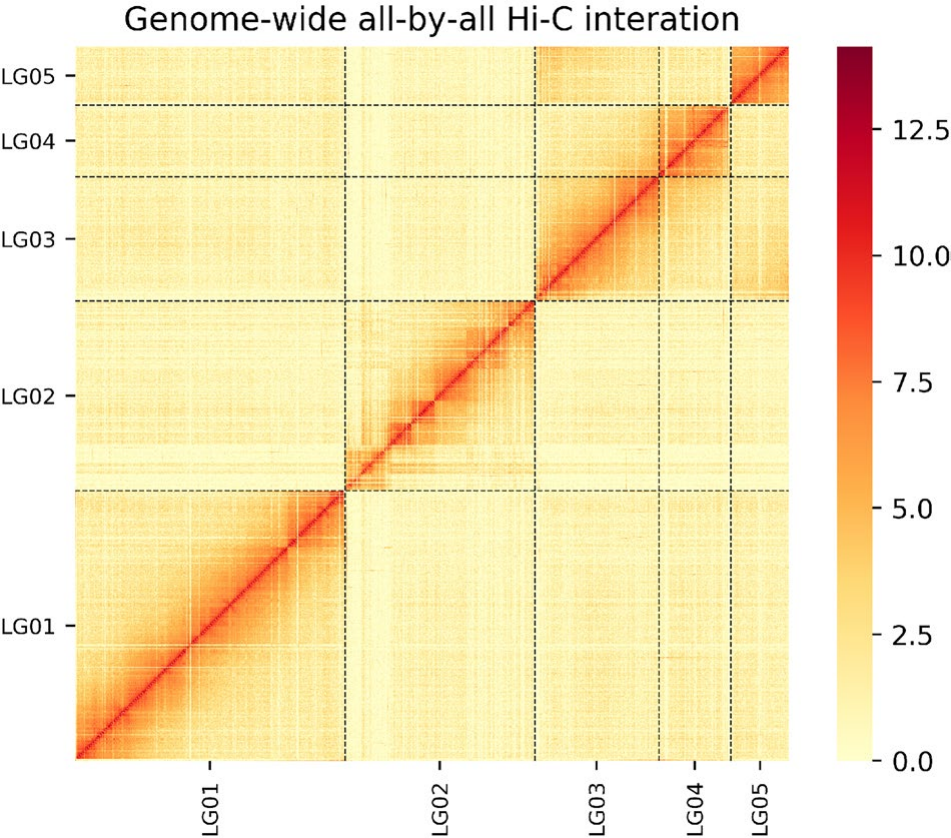
Heatmap of genome-wide Hi-C data. The heatmap of chromosome interactions in *M. crassicauda*. The frequency of Hi-C interaction links is represented by colors, which ranges from yellow (low) to red (high).

**Fig. 2:**
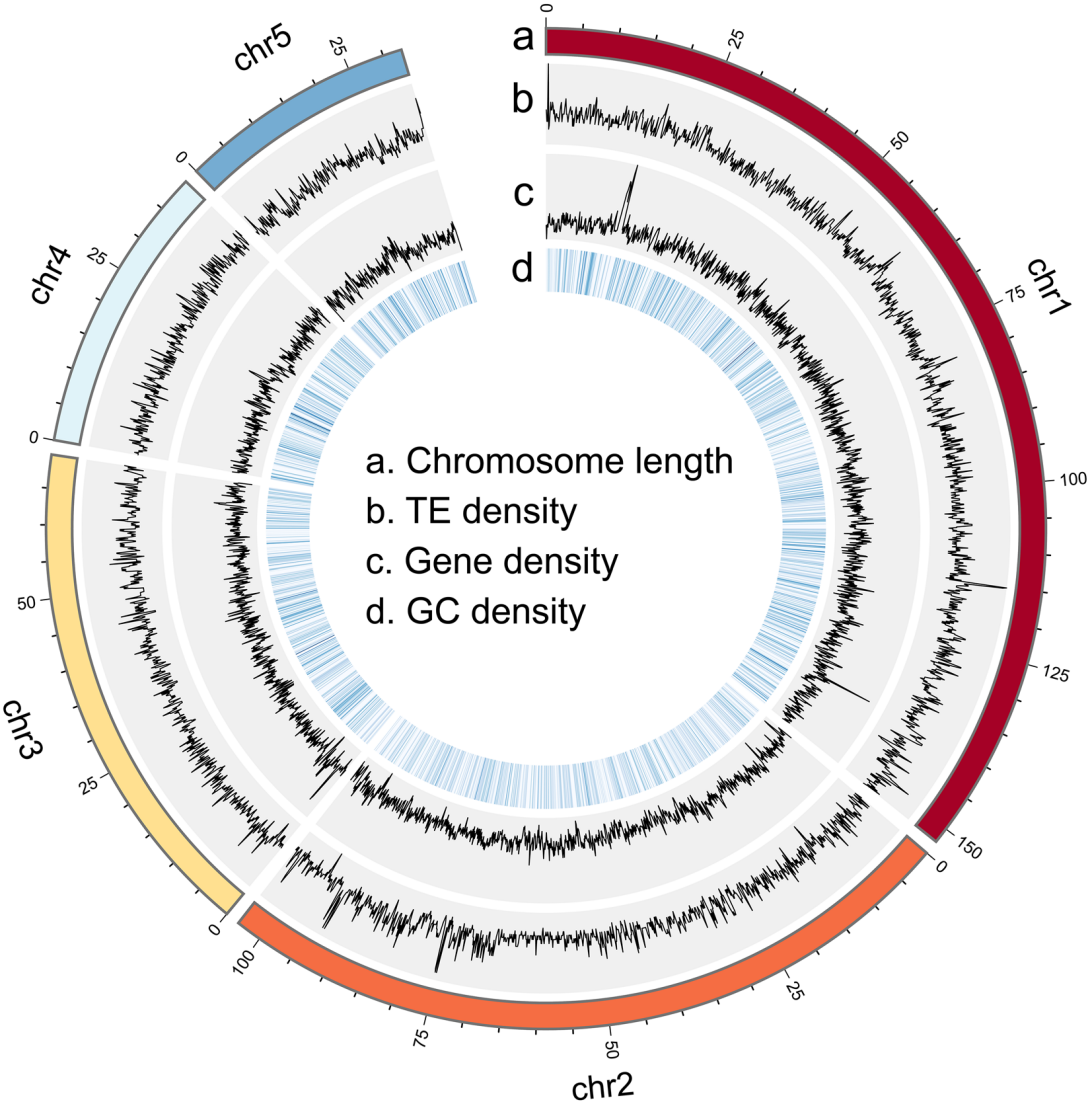
Circos plot of distribution of the genomic elements in *M. crassicauda*. The tracks indicate (**a**) length of the chromosome (Mb), (**b**) distribution of transposable element (TE) density ranges from 1 to 337, (**c**) gene density ranges from 0 to 21, and (**d**) GC density ranges from 21 to 56. The densities of TEs, genes, and GC were calculated in 100 kb windows.

By integrating Hi-C data with the contig-level assembly, a chromosome-scale assembly was constructed, consisting of five large scaffolds (Fig. 1a), aligning with the documented haploid chromosome count for this species^26^. This scaffolding process successfully anchored 93.74% of contigs into chromosome-level sequences, yielding a scaffold N50 of 103.55 Mb (Table 1). The chromosomal sizes ranged from 32.61 Mb (smallest) to 152.17 Mb (largest), with complete size distribution detailed in Table 2 and Fig. 2a.

**Table 2:** Summary of the assembled five chromosomes of *Megoura crassicauda*.

Chromosome	Size (bp)	Contig Number
LG01	152,167,074	50
LG02	103,542,494	95
LG03	69,557,264	28
LG04	39,874,475	29
LG05	32,608,204	11
Total	397,749,511	213

### Repeat and non-coding RNA annotation

Tandem repeat elements annotation was performed using Tandem Repeat Finder v4.07b (parameters: 2 7 7 80 10 50 500 -f -d -h -r)^27^. For the detection of transposable elements (TEs), a dual-method approach was employed. First, a *de novo* repeat library was constructed using RepeatModeler v1.0.11 and MITE-Hunter^28^ with default settings. This library was searched against the Repbase^29^ to classify repeat families using RepeatMasker v1.331, and the results were merged with Repbase to create a comprehensive repeat sequence library. Subsequently, RepeatMasker v1.331 was applied to predict TEs based on this final TE library. The analysis revealed that repeat sequences constitute 29.80% of the genome, with TEs accounting for the majority (26.54%) (Table 3 and Fig. 2b).

**Table 3:** Statistics of the repeat elements in *Megoura crassicauda* genome.

Type		Number of elements	Length of sequence (bp)	Percentage of sequence (%)
TEs	LINE	54,417	17,768,736	4.19
	LTR	32,820	10,377,301	2.45
	SINE	4,586	538,148	0.13
	DNA	322,184	70,192,291	16.54
	MITE	58,296	11,821,572	2.79
	RC	8,971	1,884,494	0.44
Tandem Repeats	SSR	79,509	1,002,253	0.24
	Tandem repeat elements	32,027	2,443,373	0.58
Unknown		23,696	8,344,127	1.97
Other		14,061	1,975,306	0.47
Simple repeats		1,106	100,905	0.02
Low complexity		9	1,421	0.00
Total repeats		631,682	126,449,927	29.80

Non-coding RNAs (ncRNAs), encompassing ribosomal RNAs (rRNAs), small nuclear RNAs (snRNAs) and microRNAs (miRNAs), were identified by aligning the assembled genome sequences to the Rfam database^30^ using Infernal v1.1.2^31^ with default settings. RNAmmer v1.2^32^ was employed to predict rRNA subunits, while tRNAscan-SE v2.0^33^ was utilized for the identification of transfer RNAs (tRNAs). The analyses identified 72 rRNAs, 165 small RNAs, 352 regulatory RNAs, and 370 tRNAs (Table 4).

**Table 4:** Summary of non-coding RNAs predicted within the genome of *Megoura crassicauda*.

Type		Copy number	Average length (bp)	Total length (bp)	Percentage of sequence (%)
rRNA	18S	8	2,487.00	19,896	0.0047
	28S	8	4,732.38	37,859	0.0089
	5.8S	2	158.00	316	0.0001
	5S	54	114.96	6,208	0.0015
miRNA	snRNA	34	102.68	3,491	0.0008
	miRNA	58	87.26	5,061	0.0012
	spliceosomal	62	158.94	9,854	0.0023
	other	11	173.55	1,909	0.0004
Regulatory	cis-regulatory elements	352	46.80	16,474	0.0039
tRNA	tRNA	370	75.28	27,855	0.0066

### Protein coding gene prediction and functional annotation

The identification of gene models from the TE soft-masked *M. crassicauda* genome was performed through a comprehensive strategy that combined transcriptomic evidence, *ab initio*, and homology-driven approaches. For transcriptome-based analysis, clean reads were mapped to the assembled genome using STAR v2.7.3a^34^ with the standard parameters. Subsequently, StringTie v1.3.4d^35^ was employed to obtain transcript locations, followed by open reading frame prediction via PASA v2.3.3^36^.

For *ab initio* prediction, the PASA-derived transcript dataset served as input for GeneMark-ST v5.1^37^ to establish a self-training framework, which was then applied to Augustus v3.3.1^38^ for gene model inference. In the homology-based approach, protein sequences from three well-annotated aphid genomes (consisting of *A. pisum*, *Rhopalosiphum maidis* and *Sitobion miscanthi*) were aligned to the *M. crassicauda* genome using GeMoMa v1.6.1^39^. The outputs from these three methodologies were integrated into a unified gene model set using EvidenceModeler v1.1.1, with parameters set to -segmentSize 1,000,000 and -overlapSize 100,000^36^. This process yielded a final set of 14,717 protein-coding gene models, characterized by an average gene length of 9.81 kb, average coding sequence length of 1.41 kb, average exon number of 6.46, average exon length of 217.81 bp, and average intron length of 1.54 kb. The gene density in genome assembly is shown in Fig. 2c.

To elucidate the functional characteristics of the predicted genes, protein sequences encoded by the predicted gene models were aligned to four databases: the non-redundant protein database (nr), Swiss-Prot, Kyoto Encyclopedia of Genes and Genomes (KEGG)^40^, and eukaryotic orthologous groups (KOG) databases^41^ using BLASTP v2.7.1 with a E-value threshold of 1e-5. Additionally, InterProScan v5.32-71.0^42^ was utilized to assign Gene Ontology (GO) terms for protein domain annotation. In total, 14,071 genes were functionally annotated, representing for 95.61% of the total predicted gene models (Table 5).

**Table 5:** Summary of functional annotation of protein-coding genes in the genome of *Megoura crassicauda*.

Type		Number	Percent (%)
Annotation	Swiss-Prot	10,201	69.31
	KEGG	6,376	43.32
	KOG	8,394	57.04
	GO	7,706	52.36
	NR	13,418	91.17
Total	Annotated	14,071	95.61
	Gene	14,717	—

### Phylogeny

To investigate the phylogeny among *M. crassicauda* and other aphid species, we conducted a comparative genomic analysis using the longest predicted protein sequences from 12 aphid genomes: *A. glycines*^11^, *A. gossypii*^12^, *A. pisum*^9^, *Cinara cedri*^43^, *Drepanosiphum platanoidis*^44^, *Eriosoma lanigerum*^45^, *Hormaphis cornu*^46^, *M. perisicae*^9^, *R. maidis*^47^, *Rhopalosiphum padi*^48^, *Therioaphis trifolii*^49^, *M. crassicauda*, and *Apolygus lucorum*^13^ was used as an outgroup. Orthologous groups were resolved using OrthoFinder v2.5.4^50^, identifying 1,899 single-copy orthogroups.

**Fig. 3:**
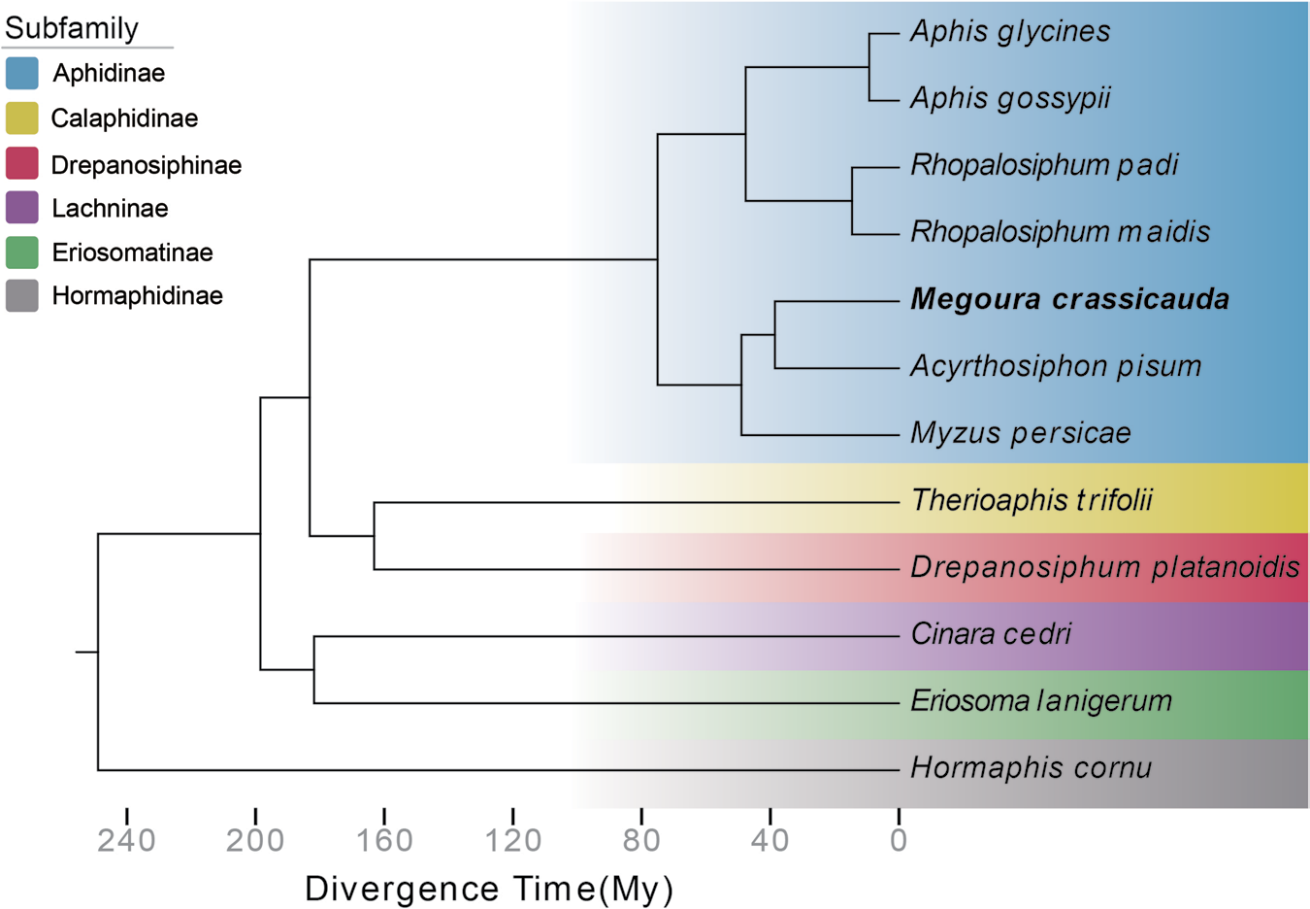
Phylogeny between *M. crassicauda* and other aphid species. The phylogenetic tree was constructed based on 1,899 single-copy orthogroups obtained from the genomes of all tested aphids. Hemiptera species *Apolygus lucorum* (not shown) was selected as the outgroup. Aphid species are colored according to their subfamily.

These conserved orthologs were concatenated to construct a multiple sequence alignment for phylogenetic inference. The species tree of the 12 aphids was generated using ORTHOFINDER v2.5.4 and rooted by STRIDE^51^ with the designated outgroup. Divergence times were estimated using r8s^52^, incorporating divergence information from TimeTree (http://www.timetree.org/): *A. pisum* vs *M. persicae* 42.5-48.0 million years ago (mya) (Fig. 3).

## Data Records

The genome sequencing, RNA sequencing reads data has been updated to the National Center for Biotechnology Information (NCBI) as a BioProject no. PRJNA1227218^53^. Pacbio, Hi-C, Illumina and transcriptome sequencing reads have been deposited in the Sequence Read Archive (SRA) databases with the accession number of SRP566327^54^. Genome assembly has been deposited at GenBank under the accession JBMIHI000000000.1^55^, and is also available at Zenodo^56^. The gene sequences annotated from the genome assembly is available at Zenodo^57^.

## Technical Validation

The validation of the contig assembly’s accuracy and completeness was conducted through three distinct approaches. First, clean Illumina reads were aligned against the contigs assembled using BWA v0.7.12, and SAMtools v1.4^58^ was utilized to determine both the total number of mapped reads and the overall mapping efficiency, which achieved an impressive rate of 97.27%. Second, Benchmarking Universal Single-Copy Orthologs (BUSCO) v4.0.5^59^ was employed to evaluate the completeness of the genome assembly based on the arthropoda_odb9 database (-l arthropoda_odb9 -m genome), the BUSCO analysis indicated that 97.75% of gene orthologs were identified in *M. crassicauda*, including single-copy and duplicated BUSCOs of 93.81% and 3.94%, respectively. Finally, CEGMA v2^60^ with default parameters was used to verify the integrity of the core genes within the assembly, 243 core eukaryotic genes were assembled, with 97.98% classified as complete.

The integrity of the annotated gene set was verified through two validation methods. Firstly, the BUSCO analysis was performed using the arthropoda_odb9 database (-l arthropoda_odb9 -m prot) to assess gene completeness. The results indicated that 93.35% of the complete ortholog genes, including 88.75% of complete and single-copy BUSCOs and 4.60% complete and duplicated BUSCOs, were present in the annotated protein set. Secondly, gene expression analysis was conducted using RNA-Seq reads from *M. crassicauda* transcriptomes. The analysis revealed that 12,306 (83.62%) annotated genes were expressed in at least one transcriptomic sample.

## Data Availability

No custom scripts or codes were used in this study. The software and pipelines mentioned above were executed with default parameters unless specifically indicated.

## References

[1] Graham, P. H. & Vance, C. P. Legumes: Importance and Constraints to Greater Use. *Plant Physiology* **131**, 872–877 (2003). 12644639 10.1104/pp.017004 PMC1540286

[2] Sharma, H. C., Srivastava, C. P., Durairaj, C. & Gowda, C. L. L. in *Climate Change and Management of Cool Season Grain Legume Crops* (eds Shyam Singh Yadav & Robert Redden) 115-139 (Springer Netherlands, 2010).

[3] Takemura, M., Nishida, R., Mori, N. & Kuwahara, Y. Acylated flavonol glycosides as probing stimulants of a bean aphid, *Megoura crassicauda*, from Vicia angustifolia. *Phytochemistry* **61**, 135–140 (2002). 12169306 10.1016/s0031-9422(02)00226-1

[4] Blackman, R. L. & Eastop, V. F. *Aphids on the world’s crops*. (John Wiley and Sons, Chichester, 2000).

[5] Hales, D. F., Gillespie, P. S., Wade, S. & Dominiak, B. C. First detection of “*Megoura crassicauda*” Maudvilko (Hemiptera: Aphididae) in Australia and a review of its biology. *General and Applied Entomology: The Journal of the Entomological Society of New South Wales* **45**, 77–81 (2017).

[6] Pickett, J. A. & Griffiths, D. C. Composition of aphid alarm pheromones. *Journal of Chemical Ecology* **6**, 349–360 (1980).

[7] Wang, B., Jacquin-Joly, E. & Wang, G. The Role of (E)-β-Farnesene in Tritrophic Interactions: Biosynthesis, Chemoreception, and Evolution. *Annu Rev Entomol* **70**, 313–335 (2025). 39378330 10.1146/annurev-ento-013024-021018

[8] Song, X., Qin, Y. G., Yin, Y. & Li, Z. X. Identification and Behavioral Assays of Alarm Pheromone in the Vetch Aphid *Megoura viciae*. *J Chem Ecol* **47**, 740–746 (2021). 34347235 10.1007/s10886-021-01297-4

[9] Mathers, T. C. *et al*. Chromosome-Scale Genome Assemblies of Aphids Reveal Extensively Rearranged Autosomes and Long-Term Conservation of the X Chromosome. *Mol Biol Evol* **38**, 856–875 (2021). 32966576 10.1093/molbev/msaa246 PMC7947777

[10] Mei, Y. *et al*. InsectBase 2.0: a comprehensive gene resource for insects. *Nucleic Acids Res* **50**, D1040–D1045 (2021). 10.1093/nar/gkab1090 PMC8728184 34792158

[11] Mathers, T. C. Improved Genome Assembly and Annotation of the Soybean Aphid (Aphis glycines Matsumura). *G3 (Bethesda, Md.)* **10**, 899–906 (2020). 31969427 10.1534/g3.119.400954 PMC7056979

[12] Zhang, S. *et al*. Chromosome-level genome assemblies of two cotton-melon aphid Aphis gossypii biotypes unveil mechanisms of host adaption. *Mol Ecol Resour* **22**, 1120–1134 (2022). 34601821 10.1111/1755-0998.13521

[13] Liu, Y. *et al*. Apolygus lucorum genome provides insights into omnivorousness and mesophyll feeding. *Mol Ecol Resour* (2020). 10.1111/1755-0998.13253 32939994

[14] Huang, T. *et al*. Identification and functional characterization of ApisOr23 in pea aphid *Acyrthosiphon pisum*. *J Integr Agr* **21**, 1414–1423 (2022).

[15] Wang, B. *et al*. A conserved odorant receptor identified from antennal transcriptome of *Megoura crassicauda* that specifically responds to *cis*-jasmone. *J Integr Agr* **21**, 2042–2054 (2023).

[16] Chen, S., Zhou, Y., Chen, Y. & Gu, J. fastp: an ultra-fast all-in-one FASTQ preprocessor. *Bioinformatics* **34**, i884–i890 (2018). 30423086 10.1093/bioinformatics/bty560 PMC6129281

[17] Marçais, G. & Kingsford, C. A fast, lock-free approach for efficient parallel counting of occurrences of k-mers. *Bioinformatics* **27**, 764–770 (2011). 21217122 10.1093/bioinformatics/btr011 PMC3051319

[18] Vurture, G. W. *et al*. GenomeScope: fast reference-free genome profiling from short reads. *Bioinformatics* **33**, 2202–2204 (2017). 28369201 10.1093/bioinformatics/btx153 PMC5870704

[19] Liu, H., Wu, S., Li, A. & Ruan, J. SMARTdenovo: a de novo assembler using long noisy reads. *Gigabyte* **1**, gigabyte15 (2021). 10.46471/gigabyte.15 PMC9632051 36824332

[20] Chaisson, M. J. & Tesler, G. Mapping single molecule sequencing reads using basic local alignment with successive refinement (BLASR): application and theory. *BMC Bioinformatics* **13**, 238 (2012). 22988817 10.1186/1471-2105-13-238 PMC3572422

[21] Li, H. & Durbin, R. Fast and accurate short read alignment with Burrows-Wheeler transform. *Bioinformatics* **25**, 1754–1760 (2009). 19451168 10.1093/bioinformatics/btp324 PMC2705234

[22] Hu, J., Fan, J., Sun, Z. & Liu, S. NextPolish: a fast and efficient genome polishing tool for long-read assembly. *Bioinformatics* **36**, 2253–2255 (2020). 31778144 10.1093/bioinformatics/btz891

[23] Langmead, B. & Salzberg, S. L. Fast gapped-read alignment with Bowtie 2. *Nat Methods* **9**, 357–359 (2012). 22388286 10.1038/nmeth.1923 PMC3322381

[24] Servant, N. *et al*. HiC-Pro: an optimized and flexible pipeline for Hi-C data processing. *Genome Biol* **16**, 259 (2015). 26619908 10.1186/s13059-015-0831-x PMC4665391

[25] Burton, J. N. *et al*. Chromosome-scale scaffolding of de novo genome assemblies based on chromatin interactions. *Nat Biotechnol* **31**, 1119–1125 (2013). 24185095 10.1038/nbt.2727 PMC4117202

[26] Blackman, R. L. & Eastop, V. F. *Aphids on the world’s crops* (2020).

[27] Benson, G. Tandem repeats finder: a program to analyze DNA sequences. *Nucleic Acids Res* **27**, 573–580 (1999). 9862982 10.1093/nar/27.2.573 PMC148217

[28] Han, Y. & Wessler, S. R. MITE-Hunter: a program for discovering miniature inverted-repeat transposable elements from genomic sequences. *Nucleic Acids Res* **38**, e199 (2010). 20880995 10.1093/nar/gkq862 PMC3001096

[29] Bao, W., Kojima, K. K. & Kohany, O. Repbase Update, a database of repetitive elements in eukaryotic genomes. *Mob DNA* **6**, 11 (2015). 26045719 10.1186/s13100-015-0041-9 PMC4455052

[30] Griffiths-Jones, S. *et al*. Rfam: annotating non-coding RNAs in complete genomes. *Nucleic Acids Res* **33**, D121–124 (2005). 15608160 10.1093/nar/gki081 PMC540035

[31] Nawrocki, E. P. & Eddy, S. R. Infernal 1.1: 100-fold faster RNA homology searches. *Bioinformatics* **29**, 2933–2935 (2013). 24008419 10.1093/bioinformatics/btt509 PMC3810854

[32] Lagesen, K. *et al*. RNAmmer: consistent and rapid annotation of ribosomal RNA genes. *Nucleic Acids Res* **35**, 3100–3108 (2007). 17452365 10.1093/nar/gkm160 PMC1888812

[33] Lowe, T. M. & Eddy, S. R. tRNAscan-SE: a program for improved detection of transfer RNA genes in genomic sequence. *Nucleic Acids Res* **25**, 955–964 (1997). 9023104 10.1093/nar/25.5.955 PMC146525

[34] Dobin, A. *et al*. STAR: ultrafast universal RNA-seq aligner. *Bioinformatics* **29**, 15–21 (2013). 23104886 10.1093/bioinformatics/bts635 PMC3530905

[35] Kovaka, S. *et al*. Transcriptome assembly from long-read RNA-seq alignments with StringTie2. *Genome Biol* **20**, 278 (2019). 31842956 10.1186/s13059-019-1910-1 PMC6912988

[36] Haas, B. J. *et al*. Automated eukaryotic gene structure annotation using EVidenceModeler and the Program to Assemble Spliced Alignments. *Genome Biol* **9**, R7 (2008). 18190707 10.1186/gb-2008-9-1-r7 PMC2395244

[37] Tang, S., Lomsadze, A. & Borodovsky, M. Identification of protein coding regions in RNA transcripts. *Nucleic acids research* **43**, e78–e78 (2015). 25870408 10.1093/nar/gkv227 PMC4499116

[38] Stanke, M., Diekhans, M., Baertsch, R. & Haussler, D. Using native and syntenically mapped cDNA alignments to improve de novo gene finding. *Bioinformatics* **24**, 637–644 (2008). 18218656 10.1093/bioinformatics/btn013

[39] Keilwagen, J. *et al*. Using intron position conservation for homology-based gene prediction. *Nucleic Acids Res* **44**, e89 (2016). 26893356 10.1093/nar/gkw092 PMC4872089

[40] Kanehisa, M. & Goto, S. KEGG: kyoto encyclopedia of genes and genomes. *Nucleic Acids Res* **28**, 27–30 (2000). 10592173 10.1093/nar/28.1.27 PMC102409

[41] Galperin, M. Y., Makarova, K. S., Wolf, Y. I. & Koonin, E. V. Expanded microbial genome coverage and improved protein family annotation in the COG database. *Nucleic Acids Res* **43**, D261–269 (2015). 25428365 10.1093/nar/gku1223 PMC4383993

[42] Zdobnov, E. M. & Apweiler, R. InterProScan–an integration platform for the signature-recognition methods in InterPro. *Bioinformatics* **17**, 847–848 (2001). 11590104 10.1093/bioinformatics/17.9.847

[43] Julca, I. *et al*. Phylogenomics Identifies an Ancestral Burst of Gene Duplications Predating the Diversification of Aphidomorpha. *Mol Biol Evol* **37**, 730–756 (2020). 31702774 10.1093/molbev/msz261 PMC7038657

[44] Crowley, L. M. & James, R. The genome sequence of the Common Sycamore Aphid, *Drepanosiphum platanoidis* (Schrank, 1801). *Wellcome Open Res* **8**, 481 (2023). 39193089 10.12688/wellcomeopenres.20169.1 PMC11347910

[45] Biello, R. *et al*. A chromosome-level genome assembly of the woolly apple aphid, *Eriosoma lanigerum* Hausmann (Hemiptera: Aphididae). *Mol Ecol Resour* **21**, 316–326 (2021). 32985768 10.1111/1755-0998.13258

[46] Korgaonkar, A. *et al*. A novel family of secreted insect proteins linked to plant gall development. *Curr Biol* **31**, 1836–1849.e1812 (2021). 33657407 10.1016/j.cub.2021.01.104 PMC8119383

[47] Chen, W. *et al*. Genome sequence of the corn leaf aphid (*Rhopalosiphum maidis* Fitch). *GigaScience* **8** (2019). 10.1093/gigascience/giz033 PMC6451198 30953568

[48] Thorpe, P., Escudero-Martinez, C. M., Cock, P. J. A., Eves-van den Akker, S. & Bos, J. I. B. Shared Transcriptional Control and Disparate Gain and Loss of Aphid Parasitism Genes. *Genome Biol Evol* **10**, 2716–2733 (2018). 30165560 10.1093/gbe/evy183 PMC6186164

[49] Huang, T. *et al*. Chromosome-level genome assembly of the spotted alfalfa aphid *Therioaphis trifolii*. *Sci Data* **10**, 274 (2023). 37173339 10.1038/s41597-023-02179-y PMC10181989

[50] Emms, D. M. & Kelly, S. OrthoFinder: phylogenetic orthology inference for comparative genomics. *Genome Biol* **20**, 238 (2019). 31727128 10.1186/s13059-019-1832-y PMC6857279

[51] Emms, D. M. & Kelly, S. STRIDE: Species Tree Root Inference from Gene Duplication Events. *Mol Biol Evol* **34**, 3267–3278 (2017). 29029342 10.1093/molbev/msx259 PMC5850722

[52] Sanderson, M. J. Estimating absolute rates of molecular evolution and divergence times: a penalized likelihood approach. *Mol Biol Evol* **19**, 101–109 (2002). 11752195 10.1093/oxfordjournals.molbev.a003974

[53] Huang, T. The raw reads and genome assembly of *Megoura crassicauda*. *ENA European Nucleotide Archive* https://identifiers.org/ena.embl:PRJNA1227218 (2025).

[54] *NCBI Sequence Read Archive* https://identifiers.org/insdc.sra:SRP566327 (2025).

[55] Huang, T. Megoura crassicauda isolate TH-2025a, whole genome shotgun sequencing project. Genbank https://identifiers.org/ncbi/insdc:JBMIHI000000000.1 (2 (2025).

[56] Huang, T. *et al*. Chromosome-level genome assembly of *Megoura crassicauda*. *Zenodo*10.5281/zenodo.15034695 (2025).

[57] Huang, T. *et al*. Annotation results of the chromosome-level genome assembly of *Megoura crassicauda*. *Zenodo*10.5281/zenodo.15034741 (2025).

[58] Li, H. *et al*. The Sequence Alignment/Map format and SAMtools. *Bioinformatics* **25**, 2078–2079 (2009). 19505943 10.1093/bioinformatics/btp352 PMC2723002

[59] Simão, F. A., Waterhouse, R. M., Ioannidis, P., Kriventseva, E. V. & Zdobnov, E. M. BUSCO: assessing genome assembly and annotation completeness with single-copy orthologs. *Bioinformatics* **31**, 3210–3212 (2015). 26059717 10.1093/bioinformatics/btv351

[60] Parra, G., Bradnam, K. & Korf, I. CEGMA: a pipeline to accurately annotate core genes in eukaryotic genomes. *Bioinformatics* **23**, 1061–1067 (2007). 17332020 10.1093/bioinformatics/btm071

